# The role of classroom- and individual-level teen stereotypes in Chinese adolescents' academic adjustment: A multilevel analysis

**DOI:** 10.3389/fpsyg.2022.933485

**Published:** 2022-11-25

**Authors:** Yang Qu, Varun Devakonda, Zeyi Shi, Beiming Yang, Qian Wang

**Affiliations:** ^1^School of Education and Social Policy, Northwestern University, Evanston, IL, United States; ^2^Department of Psychology, The Chinese University of Hong Kong, Hong Kong, China

**Keywords:** adolescence, academic adjustment, class norms, multilevel analysis, stereotypes

## Abstract

Adolescence is often portrayed in a negative light in Western culture, with teens being viewed as rebellious and irresponsible. Yet, there is substantial cultural and individual variability in views of teens. The empirical research to date is limited in that it mainly examines whether teen stereotypes are influential at the individual level. Teen stereotypes might also be perpetuated at the classroom level, which may have important implications for adolescent adjustment over time. Focusing on adolescents in Chinese culture where the teen years are often viewed in a positive light, this two-wave longitudinal study employed multi-level analyses to investigate whether stereotypes of adolescence at the classroom level play a role in Chinese adolescents' academic adjustment over time (*N* = 785; 55% girls; mean age = 12.96 years). Consistent with prior research on views of teens, the present analyses suggested that teen stereotypes regarding family obligation and school engagement at the individual level predicted adolescents' value of school and self-regulated learning strategies over the seventh grade. More importantly, classroom-level teen stereotypes were also largely predictive of adolescents' value of school and self-regulated learning strategies over time, controlling for their earlier academic adjustment, individual-level teen stereotypes, and classroom-level adjustment. Taken together, these findings indicate that stereotypes of adolescence in classroom or peer settings may contribute to adolescents' academic adjustment during this phase. The findings also provide a potential foundation for interventions aimed at promoting adolescents' positive development via changing teen stereotypes in the classroom.

## Introduction

Stereotypes of adolescent “storm and stress” are prominent in Western cultures, as evidenced by the fact that many people expect normative adolescent development to consist of irresponsible and rebellious behavior (e.g., Buchanan and Holmbeck, 1998; Buchanan, 2003). The pioneering work by Buchanan and colleagues has suggested that the pervasiveness of such stereotypes may play a role in shaping adolescents' developmental outcomes (e.g., Buchanan and Hughes, 2009). However, the degree to which individuals hold these stereotypes varies across cultures (e.g., Whiteman and Buchanan, 2002; Qu et al., 2016). For example, Chinese adolescents tend to view the teen years as a time of fulfilling family obligations or engaging in school, and such positive views are related to adolescents' improved academic and psychological adjustment over time (Qu et al., 2016, 2018, 2020a). Importantly, although prior research has highlighted how teen stereotypes can influence adolescent behavior, all of this work has focused on the role of stereotypes at the individual level. Given that peers groups (e.g., classmates) become increasingly important in socializing one's behaviors and values during adolescence (e.g., Kindermann, 1993; Ryan, 2001; Chen et al., 2008), it is possible that teen stereotypes may also be perpetuated at the classroom level, which may carry significant implications for adolescents' school adjustment over time. As a consequence, adolescents' adjustment may be influenced not only by their individual-level stereotypical beliefs about adolescence, but also by stereotypes that are shared in the classroom (Wolff, 2021). It may be particularly true for Chinese adolescents because Chinese culture highly values the role of peer groups (e.g., classmates) in promoting socially acceptable behaviors and values (Chen, 2000). Therefore, the goal of the present study was to utilize a multi-level approach to investigate whether teen stereotypes at the classroom level play a role in Chinese adolescents' adjustment over time. In doing so, the present study aims to highlight the important role of environment in perpetuating positive views of adolescence (i.e., instead of the “storm and stress” characterization), as well as contribute to ongoing efforts to reconceptualize the modern understanding of adolescence.

### Teen stereotypes in Western and Chinese cultures

Stereotypes are conceptualized as shared beliefs about the kinds of traits that characterize members of a social group or category (Greenwald and Banaji, 1995). Stereotypes are used to make generalizations about various groups of people based on salient attributes such as gender, race, and age; however, these generalizations can often affirm inaccurate or exaggerated beliefs (e.g., Steele, 1997; Hudley and Graham, 2001; Nasir et al., 2017). A particularly prominent social category that frequently serves as the basis for stereotypes is the developmental phase that a person belongs to (e.g., adolescence; Buchanan and Holmbeck, 1998). Prior research has shown that adolescents in Western contexts are often viewed as being irresponsible, rebellious, and prone to “storm and stress” (e.g., Buchanan and Holmbeck, 1998; Buchanan and Bruton, 2016). However, such Western views of the teen years may not be shared uniformly across cultures.

Adolescence in China is largely viewed as being centered around fulfilling filial piety (Qu et al., 2016). Filial piety includes, among other things, children repaying the family for their efforts in raising them, bringing honor to the family, making sacrifices for the family, and supporting the family materially as well as psychologically (Ho, 1996; Chao and Tseng, 2002). As Chinese children progress toward adolescence, which is an initial step toward maturity, they may generally believe that this period is largely marked with taking family responsibilities and meeting parents' expectations so as to repay their parents, unlike their Western counterparts who believe that doing so may interfere with their establishment of independence in this period (Pomerantz et al., 2011). Moreover, a key way to fulfill responsibilities to parents is in part through children's heightened engagement in school and improved academic achievement (Chao, 2000; Pomerantz et al., 2000; Qu and Pomerantz, 2015). On the one hand, working hard in school carries a moral value that marks one's virtue of diligence and self-perfection (Li, 2005), which brings honor to the family and fulfills filial piety (Chao, 2000; Ng and Wei, 2020). On the other hand, school engagement and good academic achievement also have strong practical values in Chinese societies because outperforming others in competitive public examinations (e.g., high school or college entrance examinations) could determine one's upward mobility and bring financial returns to the family in adulthood (Ng and Wei, 2020). As a result, Chinese adolescents may view the teen years as a time of fulfilling family and academic responsibilities (Qu et al., 2016). This notion was further supported by prior studies which compared Chinese adolescents from Hong Kong (i.e., a former British colony and special administrative region of China) and Mainland China (Qu et al., 2020a,c). Indeed, adolescents in both regions, which are deeply rooted in the Confucian tradition, view adolescence as a period of increased family obligation and heightened or similar school engagement compared to earlier years. Therefore, the current research paid special attention to the role of teen stereotypes regarding family obligation and school engagement in adolescent development in Chinese culture.

### The role of individual-level teen stereotypes in adolescents' adjustment

As individual adolescents' own endorsement of teen stereotypes varies both within and across cultures, such individual-level teen stereotypes may underlie divergent pathways of their adjustment. During this transitionary period, adolescents experience complex changes in social, emotional, and behavioral domains (for reviews, see Collins and Steinberg, 2006; Jennings and Reingle, 2012; Lee et al., 2014). Amidst all of these significant changes, teen stereotypes may play a critical role in affecting how children navigate the teen years. Buchanan and Hughes (2009) argue that this process can occur through self-fulfilling prophecies. Specifically, if adolescents receive stereotypical messaging about what behaviors are considered normative during adolescence, this may cause them to alter their own expectations and standards. Such expectations and standards may engender behavioral changes in adolescents, as well as cause perceptual biases, in that adolescents may notice and report more of certain behaviors or beliefs that comply with the stereotypical messages than their actual experience (Buchanan and Hughes, 2009). As a result, adolescents themselves may start to hold stereotypical beliefs about teen behaviors, which may further alter their actual behaviors accordingly (Sprott et al., 2003; Werner and Nixon, 2005). Indeed, cross-cultural research has shown that among both American and Chinese adolescents, seeing the teen years as a time of fulfilling family obligation and engaging in school is predictive of greater use of self-regulated learning strategies over early adolescence (Qu et al., 2016). Moreover, adolescents who hold specific views of adolescence as a time of ignoring family responsibilities are more likely to exhibit longitudinal increases in prefrontal cortex activity during cognitive control tasks, which accompanies increases in risk-taking behavior (Qu et al., 2018). This line of research highlights how teen stereotypes at the individual level are related to various aspects of adolescents' adjustment in multiple cultural contexts.

### The role of classroom-level teen stereotypes in adolescents' adjustment

Although teen stereotypes can operate on adolescents' adjustment at the individual level, it is also possible that teen stereotypes shared in the classroom play a critical role in shaping adolescents' adjustment. During adolescence, classrooms in schools serve as important contexts for promoting social interactions among peers. These interactions may, in turn, lead adolescents to acquire classroom norms such as shared behaviors, values, or beliefs including teen stereotypes (Benner et al., 2015). It cannot be denied that peer groups also involve friendship-based and smaller groups (i.e., cliques) in which adolescents personally choose to affiliate. However, it is highly important to understand how the norms in classroom settings (e.g., the beliefs shared among classmates) are related to adolescents' adjustment (e.g., Muntoni et al., 2021; Wolff, 2021), because such norms may be less susceptible to adolescents' self-selection. This is especially true for Chinese adolescents given that they stay with the same classmates for all subjects and grades throughout middle school (Carman and Zhang, 2012). From a social learning theoretical perspective, adolescents may reinforce certain beliefs or behaviors through direct social interactions with classmates, mimic their classmates' behaviors, or conform to the shared norms in class through modeling because they find it to be socially rewarding (Bandura, 1977; Brechwald and Prinstein, 2011; Giletta et al., 2021). For example, if adolescents receive messages from their classmates suggesting that the teen years represent a period of heightened maturity, familial obligations, and school engagement, they may be motivated to work hard in school in order to gain peer acceptance (e.g., compliments from classmates or popularity in class). Chinese adolescents, in particular, may perceive a greater level of social reward when their beliefs and behaviors are in accordance with peer norms shared among classmates. This is not only because adolescence is a period during which children become increasingly oriented toward seeking peer acceptance (Ryan, 2001), but also because Chinese culture places great value on the role that peer groups (e.g., classmates) play in promoting socially acceptable behaviors or values (Chen, 2000). Therefore, classroom-level norms such as shared stereotypes of adolescence might be especially influential for Chinese adolescents.

Indeed, prior research suggests that classroom-level attitudes and beliefs may contribute to adolescents' school adjustment (e.g., Pozzoli et al., 2012). Accumulating evidence has shown that established norms at the classroom level may play a direct role in shaping children's school motivation and academic achievement (Ryan, 2001; Chang, 2004; Muntoni et al., 2021). For example, researchers have found that among high-achieving classes in which academic achievement was valued and viewed as critical, adolescents exhibited more positive academic and social outcomes (Chen et al., 2008). The contextual effects of classroom norms have also been shown on a range of adolescent outcomes in addition to academic adjustment. For example, researchers have found that perceptions of peer norms (e.g., norms among classmates) in substance use contribute to adolescents' increased drinking and smoking behavior (Ali and Dwyer, 2009; Pedersen et al., 2013; Costello and Ramo, 2017). Similarly, classroom norms that support victims of bullying are associated with students' greater defending behavior and less passive bystander behavior in response to peer bullying (Pozzoli et al., 2012).

Although researchers have previously studied how stereotypes conveyed from sources such as parents, teachers, and peers can influence individual behavior (for reviews, see Jussim and Harber, 2005; Appel and Kronberger, 2012; Spencer et al., 2016), it is less clear whether individual behaviors are also impacted by stereotypes that are shared at a group level (e.g., among classmates). In attempting to answer this question, some preliminary work has been conducted by researchers who have focused on studying how gender stereotypes at the classroom level affect students' academic outcomes. For example, when examining whether classmates' stereotypes about gender were associated with disparate reading outcomes between boys and girls, researchers discovered that at the individual level, the degree to which a student believed that girls are expected to outperform boys in reading was negatively related to boys' reading achievement. Interestingly, the degree to which these beliefs were held at the classroom level was also predictive of boys' reading outcomes (Muntoni et al., 2021). Similar studies have found that girls' math performance and academic burnout are negatively affected by the presence of traditional gender norms within classrooms, and stereotypes held by classmates influenced girls' self-concepts to a greater extent than did their individual gender stereotypes (Salikutluk and Heyne, 2017; Wolff, 2021; Zhang et al., 2021; Andersen and Smith, 2022). These findings demonstrate that stereotypes reinforced at the classroom level may be associated with adolescents' individual behavior (Wolff, 2021). However, to our knowledge, prior studies have not leveraged a multi-level approach to investigate how classroom-level (vs. individual-level) teen stereotypes are related to adolescent adjustment. Indeed, stereotypes may affect adolescents in multiple ways and beyond individuals' own endorsement of stereotypes. The classroom context might be a powerful force in perpetuating certain norms and behaviors, and such classroom-level stereotypes may play a role in adolescents' adjustment over and above their individual-level teen stereotypes.

### The present study

The empirical research to date is limited in that previous studies have only examined whether teen stereotypes are influential at the individual level. Teen stereotypes may also be perpetuated at the classroom level, which may have important implications for adolescent adjustment over time. When peers in class predominantly see adolescence as a time of being responsible, adolescents may notice these views through expectations and conversations, and thereby use these classroom norms to guide their own behavior (e.g., ultimately increase their engagement in school). Therefore, the key goal of the current research was to investigate whether teen stereotypes at the classroom level play a role in adolescents' academic adjustment over time. Using a two-wave longitudinal design, adolescents in China participated in the fall and again in the spring of their first year of middle school (i.e., seventh grade). This time period was chosen given that upon entry into adolescence, adolescents may be particularly sensitive to information about teens as they are taking on a new role and likely to be in active search of guiding information (Ruble, 1994). It was expected that teen stereotypes at both individual and classroom levels would be related to adolescents' academic adjustment over time. Importantly, we employ multi-level analyses by examining the roles of classroom- and individual-level teen stereotypes simultaneously, while controlling for potential confounding factors (e.g., prior classroom-level and individual-level academic adjustment). Moreover, given that adolescents' gender and pubertal status as well as parents' educational attainment are sometimes related to adolescents' teen stereotypes (Qu et al., 2016, 2020a,c) and academic adjustment (e.g., Duncan and Brooks-Gunn, 1997; Ablard and Lipschultz, 1998; Sirin, 2005; Martin and Steinbeck, 2017), such demographic information was also collected and included in our analyses as covariates.

## Methods

### Participants

Participants were 785 Chinese adolescents (55% girls; mean age = 12.96 years in the fall of seventh grade, *SD* = 0.46) residing in a large city in southwest China. Participants were recruited from 15 classes in two middle schools serving primarily working- and middle-class families. In terms of achievement, one school was above-average and the other was below-average based on historical admission cut-off scores in municipal-level middle school entrance exam and students' average scores in municipal-level middle school graduation exam, with some variability within each school. In this area, almost all of the population (93%) is of *Han* descent ethnically (Chongqing Municipal Bureau of Statistics, 2018). In regard to parents' educational attainment, about a quarter of participants reported that their mothers (24%) and fathers (27%) had at least a college degree; also about a quarter reported that their mothers (25%) and fathers (26%) had a high school diploma, and the remaining half reported that their mothers (51%) and fathers (47%) had less than a high school diploma. The parental educational attainment in the sample reflected the general educational attainment in Mainland China, where only 23% of adult men and 28% of adult women have a high school diploma, and 15% of adult men and women have more than a high school education (National Bureau of Statistics of China, 2016).

### Procedure

In the fall (Wave 1) and spring (Wave 2) of seventh grade, adolescents completed surveys in their classrooms at school. The instructions for each measure were read by trained homeroom teachers and guidance was provided on how to use the scales with examples. Participants then completed the individual items on their own. Attrition from Wave 1 to 2 was 2%. A set of independent *t*-tests that compared participants completing both waves to those completing only the first revealed no significant differences at Wave 1 on any of the variables examined in this study, *t*(783)s <1.60, *p*s > 0.11. The procedure of this research was approved by the Survey and Behavioral Research Ethics Committee at the Chinese University of Hong Kong.

### Measures

The measures used in this study were initially created in English. Standard translation and back-translation procedures (Brislin, 1980) were followed to generate the Chinese versions with repeated discussions among Chinese and American members of the research team to modify the wording of the items to retain the original meaning (Erkut, 2010). Linguistic factors were taken into account so that the questions were easily understandable to Chinese participants (e.g., unfamiliar and awkward terms and phrases were avoided). Chinese versions of the measures have been used in prior research with adolescents in Mainland China (Wang and Pomerantz, 2009; Qu et al., 2016, 2020a).

#### Individual-level teen stereotypes

Participants completed the measure developed by Qu et al. (2016, 2020a) to assess their stereotypes of teens in the fall of seventh grade (Wave 1). Adolescents rated the extent to which they thought a variety of behaviors and attitudes were true during vs. before the teen years (1 = *more true before teen years*, 5 = *equally true before and during teen years*, 9 = *more true during teen years*). Specifically, the items included two attributes: (1) *family obligation* (12 items, e.g., “Be a responsible member of the family” and “Be concerned with meeting obligations to parents”; α = 0.76), and (2) *school engagement* (6 items, e.g., “Put a lot of effort into school” and “Be excited about what they are learning in school”; α = 0.71). The mean of the items comprising each attribute was taken, with lower numbers indicating the attribute is viewed as more characteristic *before* the teen years and higher numbers indicating it is more characteristic *during* the teen years. By using this relative scale, the stereotypes measured were meant to highlight the attitudes and behaviors that are specific to adolescents (vs. younger children), rather than reflecting the general attributes of the broader population.

#### Value of school

During the fall and spring of seventh grade, the value adolescents placed on their academic achievement was assessed with a modified version of Pomerantz et al., 2000 measure. Participants answered (1 = *not at all important*, 7 = *very important*) two questions (e.g., “How important is it to you to do well in this subject?” and “How important is it to you to avoid doing poorly in this subject?”) for five major subjects: language arts, math, English, biology, and political science, yielding a total of ten items. The mean of the ten items was taken, with higher numbers indicating greater value of school (αs = 0.88 at Wave 1 and 0.89 at Wave 2).

#### Self-regulated learning strategies

At both waves, adolescents' self-regulated learning strategies were assessed with the 18 items from Goal Orientation and Learning Strategies Survey (Dowson and McInerney, 2004), which asks about the use of meta-cognitive strategies—that is, monitoring, planning, and regulating strategies (e.g., “If I get confused about something at school, I go back and try to figure it out.” and “I try to plan out my schoolwork as best I can”). Participants rated how true (1 = *not at all true*, 5 = *very true*) each item was of them. The mean of the 18 items was taken, with higher numbers indicating greater engagement in school (αs = 0.92 at Wave 1 and 0.94 at Wave 2).

#### Classroom-level teen stereotypes and academic adjustment

Following prior studies examining classroom norms (e.g., Chang, 2004; Pozzoli et al., 2012), classroom-level teen stereotypes at Wave 1 were calculated by averaging all adolescents' teen stereotypes within each classroom. Similarly, classroom-level academic adjustment (i.e., value or self-regulated learning strategies) at Wave 1 was also calculated by averaging all adolescents' academic adjustment within each classroom.

#### Pubertal development

Participants completed 5-item Pubertal Development Scale (PDS; Petersen et al., 1988) at Wave 1. Both boys and girls reported on growth spurt, hair growth, and skin changes (1 = *no development*, 4 = *development is complete*); boys also reported on voice change and facial hair and girls on breast development and menarche status (1 = *no*, 4 = *yes*). The mean was taken with higher numbers indicating more advanced pubertal development (α = 0.70).

### Multilevel analysis plan

The key aim of the current research was to investigate how individual- and classroom-level teen stereotypes are associated with adolescents' academic adjustment over time. To this end, main analyses were conducted with hierarchical linear modeling (HLM8; Raudenbush et al., 2019), which was designed to analyze nested data (i.e., individual-level data nested within classrooms). Adolescents' individual-level academic adjustment (i.e., value of school or self-regulated learning strategies) in the spring of seventh grade (Wave 2) was predicted by their individual-level teen stereotypes as well as academic adjustment in the fall of seventh grade (Wave 1), along with demographic covariates (i.e., adolescents' gender, pubertal status, and parents' education). Importantly, classroom-level teen stereotypes and academic adjustment at Wave 1 were also included to predict later individual-level academic adjustment. Separate models for each teen stereotype attribute (i.e., family obligation or school engagement) and each academic adjustment (i.e., value of school or self-regulated learning strategies) were conducted. Following the recommendations for accurate estimates when the level 2 cluster size is small (McNeish and Stapleton, 2016; cluster size = 15 met the minimum number of clusters), restricted maximum likelihood (REML) estimation method was used to provide less biased estimates. The general HLM models were as follows:

Level 1 model:


(1) Wave 2 individual academic adjustmentij = β0j+β1j(Wave 1 individual teen stereotypesij)+β2j(Wave 1 individual academic adjustmentij)+β3j(genderij) + β4j(pubertal statusij)+β5j(parents' educationij) + rij

Level 2 model:


(2) $$\begin{array}{l} \beta_{0j}=\gamma_{00}+\gamma_{01}(\text{Wave 1 classroom-level teen stereotypes})+\gamma_{02}\\ (\text{Wave 1 classroom-level academic adjustment})+u_{0j} \end{array}$$

## Results

### Descriptive analyses

Table 1 shows descriptive statistics and zero-order Pearson correlations among the study variables. The more adolescents viewed teens (vs. younger children) as fulfilling family obligation at Wave 1, the more they showed better academic adjustment, as reflected in greater value of school and self-regulated learning strategies, at this time and at Wave 2. Similar associations were also evident for adolescents' school engagement stereotype and academic adjustment. Girls reported greater value of school compared with boys at both waves (Wave 1: *t* = 4.36, *p* < 0.001, Cohen's *d* = 0.31; Wave 2: *t* = 4.52, *p* < 0.001, Cohen's *d* = 0.33), though such gender differences were not evident in self-regulated learning strategies. Adolescents whose parents had higher educational attainment were more likely to view the teen years as a time of being responsible in family and school and show better academic adjustment. Consistent with expectations, one-sample *t*-tests that compared the average stereotypes of adolescence to the mid-point of the scale (i.e., 5 = equally true before and during teen years) indicated that adolescents viewed the teen years as a time of heightened family obligation and school engagement compared to the earlier years, *t* = 22.10, *p* < 0.001, Cohen's *d* = 0.79, and *t* = 15.23, *p* < 0.001, Cohen's *d* = 0.54, respectively.

**Table 1:** Descriptive statistics and correlations between the variables.

	**1**	**2**	**3**	**4**	**5**	**6**	**7**	**8**	**9**
1. Wave 1 Family obligation stereotypes	–								
2. Wave 1 School engagement stereotypes	0.50^***^	–							
3. Wave 1 Value of school	0.20^***^	0.28^***^	–						
4. Wave 1 Self-regulated learning strategies	0.24^***^	0.34^***^	0.48^***^	–					
5. Wave 2 Value of school	0.20^***^	0.28^***^	0.49^***^	0.41^***^	–				
6. Wave 2 Self-regulated learning strategies	0.24^***^	0.34^***^	0.40^***^	0.67^***^	0.44^***^	–			
7. Youth's gender	0.03	−0.02	−0.15^***^	−0.07	−0.16^***^	−0.01	–		
8. Youth's pubertal status	0.01	−0.05	−0.01	−0.04	0.04	−0.07	−0.39^***^	–	
9. Parents' education	0.07^*^	0.12^**^	0.05	0.18^***^	0.13^***^	0.19^***^	0.08^*^	−0.02	–
*Mean*	5.95	5.82	5.92	3.46	5.80	3.30	0.45	2.25	0.26
*SD*	1.21	1.51	0.98	0.75	1.02	0.80	0.50	0.61	0.39
*Range*	1.17–9	1–9	1–7	1–5	1–7	1–5	0–1	1–3.6	0–1

For gender, 0 = female and 1 = male. For puberty status, 1 = no development and 4 = development is complete.

For parents' education, 0 = less than a college degree and 1 = college degree or higher.

* p < 0.05.

** p < 0.01.

*** p < 0.001.

### Individual- and classroom-level teen stereotypes and adolescents' academic adjustment

The analyses first started with a null model (i.e., intercept-only model) without explanatory variables. The intraclass correlation (ICC) of adolescents' values of school is 0.13, indicating that 13% of the total variance was explained by classroom level. With regard to adolescents' self-regulated learning strategies, the ICC showed that 7% of the total variation was allocated between classrooms. The estimated classroom variance of these two aspects of academic adjustment was also statistically significant, χ^2^(14) = 135.43, *p* < 0.001 and χ^2^(14) = 69.11, *p* < 0.001 for value of school and self-regulated learning strategies, respectively, suggesting that there were significant variations in academic adjustment across the 15 classrooms.

Next, two sets of analyses were conducted to investigate how individual- and classroom-level teen stereotypes are related to adolescents' academic adjustment over time. The first set of analyses examined the role of individual- and classroom-level teen stereotypes regarding family obligation in predicting adolescents' later academic adjustment. As shown in Table 2, with regard to adolescents' value of school, the Akaike information criterion (AIC) index showed that the model fit better than the null model (AIC = 1,943.99 vs. AIC = 2,129.78 in the null model). Family obligation stereotypes at both individual and classroom levels at Wave 1 were predictive of adolescents' greater value of school over time, *B* = 0.06, *SE* = 0.03, β = 0.07, *p* = 0.018, and *B* = 0.75, *SE* = 0.18, β = 0.17, *p* < 0.001, respectively. Individual- and classroom-level value of school at Wave 1 were also predictive of adolescents' later value, *B* = 0.43, *SE* = 0.03, β = 0.41, *p* < 0.001, and *B* = 0.29, *SE* = 0.12, β = 0.09, *p* = 0.039, respectively. Similar analyses were conducted to predict adolescents' self-regulated learning strategies over time. The AIC index showed that the model fit better than the null model (AIC = 1,380.15 vs. AIC = 1,818.65 in the null model). Consistent with findings on value of school, family obligation stereotypes at both individual and classroom levels at Wave 1 were predictive of adolescents' greater use of self-regulated learning strategies over time, *B* = 0.04, *SE* = 0.02, β = 0.06, *p* = 0.040, and *B* = 0.46, *SE* = 0.11, β = 0.13, *p* = 0.001, respectively. Adolescents' self-regulated learning strategies at the individual level, but not at the classroom level, predicted their later self-regulated learning strategies, *B* = 0.70, *SE* = 0.03, β = 0.66, *p* < 0.001.

**Table 2:** Multilevel modeling predicting academic adjustment from teen stereotypes regarding family obligation.

	**Predicting W2 value of school**				**Predicting W2 self-regulated learning strategies**		
	***B***	***SE***	**β**		***B***	***SE***	**β**
**Level 1**							
Gender	−0.18^**^	0.07	−0.09		0.02	0.05	0.01
Pubertal status	0.00	0.05	0.00		−0.05	0.04	−0.04
Parents' education	0.01	0.09	0.00		0.07	0.06	0.03
W1 individual-level teen stereotypes regarding family obligation	0.06^*^	0.03	0.07		0.04^*^	0.02	0.06
W1 individual-level value of school	0.43^***^	0.03	0.41		–	–	–
W1 individual-level self-regulated learning strategies	–	–	–		0.70^***^	0.03	0.66
**Level 2**							
W1 classroom-level teen stereotypes regarding family obligation	0.75^***^	0.18	0.17		0.46^**^	0.11	0.13
W1 classroom-level value of school	0.29^*^	0.12	0.09		–	–	–
W1 classroom-level self-regulated learning strategies	–	–	–		−0.23	0.11	−0.07
Proportion of variance at individual level explained by Level 1 variables			20.11%				62.01%
Proportion of variance at classroom level explained by Level 2 variables			99.84%				99.76%
AIC			1,943.99^a^				1,380.15^b^

For gender, 0 = female and 1 = male. For puberty status, 1 = no development and 4 = development is complete.

For parents' education, 0 = less than a college degree and 1 = college degree or higher.

AIC, akaike information criterion.

a Null model AIC = 2,129.78.

b Null model AIC = 1,818.65.

* p < 0.05.

** p < 0.01.

*** p < 0.001.

The second set of analyses investigated the role of teen stereotypes regarding school engagement at individual and classroom levels in predicting adolescents' later academic adjustment. As shown in Table 3, with regard to adolescents' value of school, the AIC index showed that the model fit better than the null model (AIC = 1,938.43 vs. AIC = 2,129.78 in the null model). The more adolescents viewed the teen years as a time of working hard in school, the more they placed greater value on school six months later, *B* = 0.08, *SE* = 0.02, β = 0.12, *p* < 0.001. However, school engagement stereotypes at the classroom level were not predictive of adolescents' greater value of school over time, *B* = 0.20, *SE* = 0.15, β = 0.08, *p* = 0.202. With regard to predicting adolescents' self-regulated learning strategies, the AIC index showed that the model fit better than the null model (AIC = 1,378.87 vs. AIC = 1,818.65 in the null model). Viewing the teen years as a time of working hard in school at the individual level predicted greater use of self-regulated learning strategies six months later, *B* = 0.05, *SE* = 0.02, β = 0.09, *p* < 0.001. Moreover, adolescents who were in a classroom where more students held this view of teens were more likely to use greater self-regulated learning strategies over time, *B* = 0.20, *SE* = 0.09, β = 0.11, *p* = 0.042.

**Table 3:** Multilevel modeling predicting academic adjustment from teen stereotypes regarding school engagement.

	**Predicting W2 value of school**				**Predicting W2 self-regulated learning strategies**		
	***B***	***SE***	**β**		***B***	***SE***	**β**
**Level 1**							
Gender	−0.17^*^	0.07	−0.08		0.03	0.05	0.02
Pubertal status	0.02	0.05	0.01		−0.04	0.04	−0.03
Parents' education	0.04	0.10	0.01		0.07	0.07	0.03
W1 individual-level teen stereotypes regarding school engagement	0.08^***^	0.02	0.12		0.05^***^	0.02	0.09
W1 individual-level value of school	0.42^***^	0.04	0.40		–	–	–
W1 individual-level self-regulated learning strategies	–	–	–		0.68^***^	0.03	0.64
**Level 2**							
W1 classroom-level teen stereotypes regarding school engagement	0.20	0.15	0.08		0.20^*^	0.09	0.11
W1 classroom-level value of school	0.41^*^	0.18	0.12		–	–	–
W1 classroom-level self-regulated learning strategies	–	–	–		−0.25	0.15	−0.07
Proportion of variance at individual level explained by Level 1 variables			21.34%				62.35%
Proportion of variance at classroom level explained by Level 2 variables			87.12%				97.60%
AIC			1,938.43^a^				1,378.87^b^

For gender, 0 = female and 1 = male. For puberty status, 1 = no development and 4 = development is complete.

For parents' education, 0 = less than a college degree and 1 = college degree or higher.

AIC, akaike information criterion.

a Null model AIC = 2,129.78.

b Null model AIC = 1,818.65.

* p < 0.05.

*** p < 0.001.

## Discussion

Western stereotypes of adolescent “storm and stress” characterize the teen years as a developmental period marked by heightened risk-taking, emotional volatility, and familial individuation (Arnett, 1999; Buchanan and Bruton, 2016). The degree to which adolescents hold such stereotypes may carry significant implications for their adjustment during the teen years; however, the applicability of these stereotypes may be limited in other cultural contexts. Indeed, prior research on Chinese adolescents has highlighted that the teen years are often viewed as a time of responsibility in Chinese culture, and adolescents who hold such positive views are more likely to exhibit positive developmental outcomes over time (Qu et al., 2016, 2020a). More importantly, teen stereotypes can operate on adolescents' adjustment not only at the individual level, but because stereotypes can be shared among peers in the classroom, stereotypes can also operate at the classroom level. For example, emerging evidence suggests that classroom-level beliefs and attitudes are associated with adolescents' academic and behavioral adjustment (e.g., Muntoni et al., 2021; Wolff, 2021). However, existing literature has not addressed whether stereotypes of adolescence at the classroom level play a unique role in shaping adolescent development, over and above the role of teen stereotypes at the individual level. Using a longitudinal design, the present study provides initial evidence that teen stereotypes at both individual and classroom level predict adolescents' academic adjustment over time.

Consistent with our expectations and other empirical findings (e.g., Qu et al., 2016, 2020a), Chinese adolescents in the current sample viewed the teen years as a period of heightened family obligation and school engagement in comparison to earlier years. The results, in contrast to Western teen stereotypes as irresponsible, rebellious, and prone to “storm and stress” (Buchanan and Bruton, 2016), may be an indication of the emphasis on the fulfillment of filial piety (e.g., meeting family obligations and having good grades to repay parents as children reaching maturity) that is highly valued in Chinese culture (e.g., Chao, 2000). More importantly, in line with our expectations, the present findings from HLM analyses suggest that classroom-level teen stereotypes regarding family obligation and school engagement were predictive of adolescents' later academic adjustment above and beyond their individual-level teen stereotypes. Notably, these analyses were stringent in terms of adjusting for earlier individual- and classroom-level adjustment, as well as a number of demographic covariates (i.e., adolescents' gender, pubertal status, parents' education). In classes where more students viewed the teen years as a time of fulfilling family obligation, adolescents were more likely to show enhanced academic adjustment in terms of value of school and self-regulated learning strategies over early adolescence. Similar results were found when more classmates viewed the teen years as a time of engaging in school, though the positive association was evident only for self-regulated learning strategies, but not value of school. These findings are in accordance with prior research which illustrates the unique role of classroom and peer settings in contributing to adolescents' academic adjustment during this developmental period (Chang, 2004; Carman and Zhang, 2012; Muntoni et al., 2021; Wolff, 2021).

Social learning that involves the process of social reinforcement may be one of the potential mechanisms through which teen stereotypes at the classroom level influence individual adolescents' academic adjustment (Bandura, 1977; Brechwald and Prinstein, 2011; Ryan et al., 2019). The social reinforcement process postulates that through social interactions with their classmates, adolescents learn what beliefs and behaviors are discouraged or encouraged among peers. For example, when the whole class generally views the teen years as a time of engaging in school, adolescents who put a lot of effort into school might be viewed favorably by their classmates, which could be socially rewarding (Foulkes and Blakemore, 2016). Moreover, shared stereotypes among classmates may influence adolescents' academic adjustment through the development of self-concepts (Wolff, 2021). Adolescents may perceive conformity to peer norms as rewarding because they often face increased pressure to be accepted within their peer groups (Brechwald and Prinstein, 2011; Giletta et al., 2021). Therefore, when adolescents conform to the shared views in their classrooms that the teen years are a time of family obligation and school engagement, they may feel especially rewarded, which may reinforce them to engage in actual behaviors in school more (e.g., greater use of self-regulated learning strategies). In contrast, when a certain teen stereotype (e.g., viewing the teen years as a time of fulfilling family obligations) is not welcomed within the classroom, adolescents who hold these beliefs or display certain behaviors (e.g., engagement in school) that comply with this stereotype might feel socially punished (i.e., rejected by peers) and experience lowered self-esteem, leading to fewer occurrences of these behaviors.

The only exception for the association between classroom-level teen stereotypes and academic adjustment is that teen stereotypes regarding school engagement at the classroom level were not predictive of adolescents' greater value of school over time. The results echo prior research, suggesting that although school engagement and family obligation are both important dimensions of teen stereotypes, they indeed capture different attributes of adolescence (e.g., Qu et al., 2016). More importantly, the results indicate that although adolescents' perceived norms may play a role in modulating their actual behaviors in school, these norms may not necessarily change their value of school. That is, classroom-level views that the teen years are a time of working hard in school may potentially guide adolescents to put more effort into school over time but may not guide them to value the importance of achieving high grades. It is possible that some adolescents' engagement in school was extrinsically motivated by peer norms, but not internalized to their own belief system. In the process of internalization of values, adolescents need to not only perceive the messages communicated by their peers but also accept these messages themselves (Grusec and Goodnow, 1994). From an optimistic perspective, this result suggests that for Chinese adolescents who do not personally place much value on school, their classroom-level school engagement teen stereotypes may at least still guide them to put more effort in school over time.

On the other side of the coin, this result may also suggest the dangerous role of classroom-level teen stereotypes in some Western cultures, which may view the teen years (vs. earlier years) as a period of heightened school disengagement (e.g., Qu et al., 2016). For example, prior research (Qu et al., 2016) found that adolescents in the U.S. (vs. China) endorsed more teen stereotypes of school disengagement, which further contributed to American adolescents' decreased engagement in school over time. Within a classroom context, it is possible that adolescents in Western cultures (e.g., the U.S.) who perceive negative classroom-level messages from peers about school engagement may also put less effort in school due to peer pressure, despite their intrinsic value of school. Understanding this may be critical for buffering or reversing the detrimental effects of negative teen stereotypes and enhancing positive teen stereotypes in different cultures. Moreover, the results should be interpreted with caution because the span of one school year in the present study may not provide adolescents with prolonged enough exposure to classroom norms. Whereas behavioral engagement in school (i.e., the use of self-regulated learning strategies) was easier to alter over a short-term period, it is possible that effectively internalizing a heightened sense of school value requires more time. Thus, although adolescents are likely to be influenced by how their classmates view adolescence with regard to school engagement and show certain behavioral changes (i.e., the use of self-regulated learning strategies), they may be more hesitant to shift the extent to which they value school.

The results were also largely aligned with our expectation as well as prior research in that individual-level teen stereotypes predicted adolescents' academic adjustment over time. Specifically, ideas that the teen years are a time of responsibility to the family and engagement in school, which were held by adolescents themselves, predicted increased value of school and self-regulated learning strategies over time. These findings are consistent with prior research on the role of teen stereotypes at the individual level in adolescents' academic, social, and emotional adjustment (e.g., Buchanan and Hughes, 2009; Qu et al., 2016, 2020c). For example, prior research suggests that individual-level teen stereotypes regarding family responsibility and school engagement predict adolescents' engagement in school over early adolescence (Qu et al., 2016). In both the United States and China, the more adolescents saw teens (vs. younger children) as responsible to the family and engaged in school, the more they used self-regulated learning strategies 6 months later over and above their earlier learning strategies. That is, these stereotypes may create adolescents' own “social realities” through self-fulfilling prophecies (Buchanan and Hughes, 2009). Taken together with the findings on classroom-level teen stereotypes, these results highlight that adolescents' individual and shared stereotypes of teens among peers may be both uniquely related to their adjustment over time.

### Theoretical and practical implications

The key findings of the current research support the notion that classroom context plays a critical role in adolescent development through shared teen stereotypes. This advances the theoretical understanding of the socialization processes of adolescent adjustment, as previous literature on teen stereotypes has focused on how societal messaging about adolescence is primarily conveyed from sources such as the media, parents, siblings, and teachers (i.e., Whiteman and Buchanan, 2002; Buchanan and Hughes, 2009). Each of these sources has the capacity to disparately influence how individuals view adolescence. Siblings, in particular, may play a vital part in shaping parents' subsequent expectations for adolescent behavior (Whiteman and Buchanan, 2002). This is especially relevant given the implications of relaxing the one-child policy in China. Moreover, while the influence of peers may be prominent, classroom-level teen stereotypes may also be largely socialized by teachers and school administrators. Therefore, additional work is needed to determine the distinct roles that these various actors play when shaping stereotypes within classroom contexts.

Furthermore, from a practical standpoint, this study sets the foundation for future experimental research aimed at promoting positive adolescent outcomes. Indeed, previous research has demonstrated the effectiveness of interventions targeting the individual level for countering adolescents' negative teen stereotypes and hence fostering constructive behavior (Qu et al., 2020b). Based on the present findings, similar interventions centered around reframing teen stereotypes at the classroom level may also prove advantageous. Rather than directly targeting individual students' stereotypic beliefs, classroom-level interventions may be more effective at changing how peers influence adolescents' personal attitudes and belief systems. These classroom-level interventions could differ from individual-level interventions in that they would likely be used to specifically target classroom context, which has not been the case in prior interventions. Working with teachers to create specific classroom-level curricula centered around promoting positive stereotypes of adolescence and peer interactions could serve as a potential avenue for shifting teen attitudes on a comprehensive scale. This could be done by engaging in collaborative activities, discussions, and role plays which aim to have students know that their classmates view adolescence in a more positive light. Similar classroom-level interventions have previously shown promise when implemented for reducing adolescents' gender stereotypes (e.g., Zhao et al., 2018). However, future research is still needed in order to develop interventions that specifically focus on teen stereotypes at the classroom level.

### Limitations and future directions

The present research has a few limitations that point to directions for future studies. First, the sample used for this study consisted of a small number of classes (i.e., 15 classes). Notably, even with such a limited sample, the present findings garnered from analyses that controlled for other confounding variables did prove fruitful in investigating the role of teen stereotypes at the classroom level in adolescent adjustment over time. However, corroboration of these findings entails a similar study across a larger number of classes to further support the notion that classroom-level stereotypes play a unique role in adolescents' academic adjustment. Moreover, due to its relatively limited scope, the current research focused only on the roles of youth's own teen stereotypes as well as their classmates' teen stereotypes. However, teen stereotypes held by parents (Buchanan, 2003) or teachers (Jussim and Eccles, 1992; Szumski and Karwowski, 2019) may also be influential on adolescents' academic adjustment. Future exploration of teen stereotypes from multiple recourses is needed to provide a refined picture of the socialization of teen stereotypes. In addition, based on the present analysis, it is unclear whether individual- and classroom-level teen stereotypes co-develop and influence each other. For example, individual- and classroom-level stereotypes of adolescence may reinforce each other over time. Therefore, future research can employ measurements across multiple time points to examine the potential covariation between individual- and classroom-level stereotypes. The current study was also lacking information about how students were assigned to each classroom. It is possible that there were selection effects in the various classrooms, such that high-achieving students were grouped together in specific classes. To take into account potential variation in academic adjustment across classrooms, classroom-level academic adjustment (i.e., value of school and self-regulated learning strategies) at Wave 1 was controlled in our analyses. Future related studies should, however, be careful to account for potential selection effects when applicable.

Second, the generalization of the findings from this study to different cultural contexts should be taken with caution, as the present sample was only comprised of Chinese adolescents. Importantly, prior research has suggested that adolescents from the United States, in contrast to Chinese adolescents, are more likely to view adolescence as a period of dampened family responsibility and school engagement, with increased peer orientation (Buchanan and Holmbeck, 1998; Qu et al., 2016). Because mainstream ideas regarding teen stereotypes can vary across cultures, this may carry implications for the kinds of messages that are reinforced within classroom settings. Therefore, a key direction for future research is to examine whether teen stereotypes at the classroom level play a similar role across cultural contexts, even in countries where adolescence is viewed in a more negative light.

Finally, although a significant strength of this study was that participants were followed longitudinally, the results are correlational. Despite incorporating a number of covariates in the analyses, causal conclusions cannot be made based on the current findings. Thus, future research which leverages an experimental approach will be beneficial for establishing a causal relationship between classroom-level teen stereotypes and adolescents' academic adjustment. A potential avenue would be to conduct interventions that specifically target stereotypes of adolescence at the classroom level, both testing theoretically causal effects of such stereotypes on adolescent adjustment and exploring practical implications for promoting academic achievement and wellbeing.

## Conclusion

The present study provides novel insights into how stereotypes of adolescence at both the individual and classroom levels are related to adolescents' academic adjustment. Using a multilevel approach, views of adolescence at the individual level regarding family obligation and school engagement—seeing the teen years as a time of fulfilling family responsibility and working hard in school—were predictive of adolescents' school value and self-regulated learning strategies over time. More importantly, classroom-level teen stereotypes were also largely predictive of youth's increased value of school and use of self-regulated learning strategies, though classroom-level stereotypes about engagement in school were not predictive of adolescents' value of school over time. In conclusion, our findings underscore that teen stereotypes in social contexts—specifically in the classroom setting—might play an important role in academic adjustment among adolescents. Shared beliefs conveyed by peer groups and classmates may potentially influence the frequency of typical adolescent behaviors, as well as the extent to which stereotypes of adolescence become engrained within society on a more collective scale. Given the prominence and impact of the “storm and stress” characterization, these results have significant implications for future research and policy-based interventions aimed at promoting, reconceptualizing, and improving adolescent development.

## Data availability statement

The raw data supporting the conclusions of this article will be made available by the authors, without undue reservation.

## Ethics statement

The studies involving human participants were reviewed and approved by the Survey and Behavioral Research Ethics Committee at the Chinese University of Hong Kong. Written informed consent to participate in this study was provided by the participants' legal guardian/next of kin.

## Author contributions

YQ and QW contributed to the conception and design of the study. QW collected the data. YQ performed the statistical analysis. YQ, VD, ZS, and BY wrote the manuscript. All authors contributed to manuscript revision, read, and approved the submitted version.

## Funding

This research is supported by Research Grants Council of Hong Kong, General Research Fund # 14659716 (to QW), US Department of Education, Institute of Education Sciences, Multidisciplinary Program in Education Sciences, Grant Award # R305B200037 (to VD), and research fund from the Center for Culture, Brain, Biology, and Learning at Northwestern University (to YQ).

## Conflict of interest

The authors declare that the research was conducted in the absence of any commercial or financial relationships that could be construed as a potential conflict of interest.

## Publisher's note

All claims expressed in this article are solely those of the authors and do not necessarily represent those of their affiliated organizations, or those of the publisher, the editors and the reviewers. Any product that may be evaluated in this article, or claim that may be made by its manufacturer, is not guaranteed or endorsed by the publisher.
